# The anti-tumor growth effect of a novel agent DMAMCL in rhabdomyosarcoma in vitro and in vivo

**DOI:** 10.1186/s13046-019-1107-1

**Published:** 2019-03-08

**Authors:** Ning Xu, Zhongyan Hua, Gen Ba, Simeng Zhang, Zhihui Liu, Carol J. Thiele, Zhijie Li

**Affiliations:** 1 Liaoning Key Laboratory of Research and Application of Animal Models for Environmental and Metabolic Diseases, Medical Research Center, Shengjing Hospital of China Medical University, Shenyang, 110004 China; 2 Cellular & Molecular Biology Section, Pediatric Oncology Branch, National Cancer Institute, National Institutes of Health, Bethesda, MD 20892 USA

**Keywords:** Rhabdomyosarcoma (RMS), DMAMCL, Vincristine (VCR), Epirubicin

## Abstract

**Background:**

Rhabdomyosarcoma (RMS) is the most common soft tissue sarcoma in children with poor survival. New treatment approaches are urgently needed to improve treatment efficacy in RMS patients. DMAMCL is a novel agent from Asteraceae family that has been tested in phase I clinical trials in adult glioma in Australia.

**Methods:**

Five RMS cell lines (RD, RH18, RH28, RH30 and RH41) were used. The in vitro anti-tumor effect of DMAMCL, alone or in combination with VCR or Epirubicin, was studied using MTS assay or IncuCyte-Zoom cell confluency assay, and further validated by xenograft-mouse model in vivo. Changes in caspase-3/7 activity, cell-cycle progression and generation of ROS after DMAMCL treatment were investigated. Bim mRNA expression was measured by RT-qPCR, and protein expressions of Bim and phosphorylated-NF-κB(p65) by Western blotting. Small interfering RNAs (siRNA) of Bim were used to study the role of Bim in DMAMCL-induced cell death.

**Results:**

In vitro, DMAMCL treatment induced a dose-dependent increase in cell death that could be blocked by pan-caspase-inhibitor-Z-VAD-fmk in five RMS cell lines. The percent of cells in SubG1 phase and activities of caspase-3/7 increased after DMAMCL treatment; The combination of DMAMCL with VCR or Epirubicin significantly increased cell death compared to each reagent alone. In vivo*,* DMAMCL(75 mg/kg or 100 mg/kg) inhibited tumor growth and prolonged survival of mice bearing xenograft RMS tumors (RD, RH18, RH30, RH41). Compared to treatment with DMAMCL or VCR, a combination of two reagents caused significant inhibition of tumor growth (RD, RH41), even after treatment termination. The expression of Bim increased at protein level after DMAMCL treatment both in vitro and in vivo. The expression of p-NF-κB(p65) had a transient increase and the generation of ROS increased after DMAMCL treatment in vitro. Transfection of Bim siRNA into RMS cells blocked the DMAMCL-induced increase of Bim and partially attenuated the DMAMCL-induced cell death.

**Conclusion:**

DMAMCL had an anti-tumor growth effect in vitro and in vivo that potentially mediated by Bim, NF-κB pathway and ROS. A combination of DMAMCL with chemotherapeutic drugs significantly increased the treatment efficacy. Our study supports further clinical evaluation of DMAMCL in combination with conventional chemotherapy.

**Electronic supplementary material:**

The online version of this article (10.1186/s13046-019-1107-1) contains supplementary material, which is available to authorized users.

## Introduction

Rhabdomyosarcoma (RMS) is a common soft tissue sarcoma which is considered relevant in the skeletal muscle lineage of childhood. It is with an incidence of 4.5 cases per 1 million children every year, which making it the third most prevalent extracranial solid tumor in children [1, 2]. RMS has four distinct histopathological subtypes: embryonal rhabdomyosarcoma (ERMS), alveolar rhabdomyosarcoma (ARMS), pleomorphic rhabdomyosarcoma, and sclerosing/spindle rhabdomyosarcoma. About 70–80% of RMS patients belong to either ERMS or ARMS [3–5]. ERMS are characterized by RAS gene mutations and account for about two thirds of RMS and have a 73% 5-year overall survival rate. ARMS are characterized by PAX/FOXO1 or related fusion oncogenes and account for about 20% of RMS and have a 48% 5-year overall survival rate [1, 6]. Recurrence, metastasis and drug resistance are the main causes of treatment failure resulting a survival rate of 40% for ERMS [7–9] and 10–30% for ARMS [9, 10] in patients with metastatic disease. Despite the development of conventional treatment, such as surgery, chemotherapy, radiotherapy, immunotherapy, the survival rate in recurrent and metastatic RMS is still less than 20% [8]. Thus more effective treatment strategies are urgently needed for the RMS patients.

VCR and Epirubicin are the chemotherapeutic agents, which are used in initial, recurrence and metastasis RMS patients in clinic [11, 12]. VCR is a chemotherapy drug which could inhibit microtubule polymerization, causes an arrest of G2/M phase, and induces cells apoptosis [11, 13]. The side effects of VCR treatment are hematologic toxicity, digestive tract toxicity and neurotoxicity. Epirubicin educes its anti-tumor effect by damaging the DNA, disturbing the synthesis of DNA, RNA, proteins, and targeting the integrity and activity of cellular membranes [14, 15], with a side effect of hematologic and dose-dependent cardiotoxicity [16]. These toxic reactions restrict the use of chemotherapeutic drugs. With the progress of disease treatment, recurrence, metastasis and drug resistance are the main causes of treatment failure in RMS patients. There is no largely change in backbone of chemotherapy [17]. So, we need to develop new agents for the treatment of cancer patients.

Parthenolide (PTL) is a natural sesquiterpene lactone (SL) of 10,5-ring structure isolated from *Tanacetum parthenium* (Feverfew) that was originally used for the treatment of inflammation in traditional Chinese medicine. Subsequently it was found to have anti-tumor growth effect, especially target on cancer stem cells. However its chemical properties limited its stability [18–21]. Micheliolide (MCL) is a guaianolide sesquiterpene lactone (GSL), which is 7 times more stable than PTL in vivo with a half-life of 2.64 h compared to 0.36 h for PTL in mouse plasma [22]. Dimethylaminomicheliolide (DMAMCL) is a pro-drug of MCL. Compared to MCL, DMAMCL has an increased stability, increased activity, and less toxicity in normal cells or normal stem cells. DMAMCL can continuously release MCL into plasma for 8 h [22], and can pass through the blood-brain barrier [23].Studies found that DMAMCL or MCL not only can inhibit inflammation (such as intestinal inflammation, hepatic steatosis [24], diabetes nephropathy [25], *Staphylococcus aureus* and MRSA infection [26], rheumatoid arthritis [27]), but also has an anti-tumor growth effect in colitis-associated cancer [28], breast cancer [29, 30] and glioma [23]. A phase I clinical trial with DMAMCL in patients with glioma is underway [23]. So far no studies with DMAMCL on RMS have been reported.

In the present study, we investigated the anti-tumor effect of DMAMCL in RMS, as a single agent or in combination with chemotherapeutic drugs in vitro and in vivo. The potential role of Bim in the DMAMCL-induced cell death was also studied.

## Materials and methods

### Cell lines and cell culture

Five human RMS cell lines (RD(ERMS, fusion negative-NRASQ61H), RH18(RMS-fusion negative), RH28(ARMS, fusion positive), RH30(ARMS, fusion positive) and RH41(ARMS, fusion positive)) and a mouse fibroblast cell line (NIH3T3) were used in this study. All cell lines were received from Dr. Carol J. Thiele (Cellular and Molecular Biology Section, Pediatric Oncology Branch, National Cancer Institute, National Institutes of Health, Bethesda, MD, USA) and determined to be genetically pure using a single-nucleotide polymorphism-based genotype assay (kindly performed by Dr. S.J. Chanock’s group in Division of Cancer Genetics and Epidemiology, NCI). The cell lines were cultured in RPMI-1640 medium (Bioind, Israel) with 10% fetal bovine serum (FBS; Bioind, Israel), 2 mM glutamine (Bioind, Israel) and antibiotics (penicillin 100 units/ml, streptomycin 100 μg/ml; Bioind, Israel) at 37 °C in 5% CO_2_ incubator.

### Reagents and antibodies

DMAMCL was a gift from Dr. Yue Chen (College of Pharmacy, The State Key Laboratory of Elemento-Organic Chemistry, and Tianjin Key Laboratory of Molecular Drug Research, Nankai University, Tianjin, China). Vincristine (VCR) was purchased from Guangdong lingnan pharmaceutical company (China), and Epirubicin was from Pfizer (America). The Reactive Oxygen Species Assay Kit was purchased from Beyotime (China). The anti-phosphorylated-NF-κB(p65) antibody (1:1000; Cell signaling, USA)(marked as p-NF-κB(p65)), anti-Bim antibody (1:1000; CST, USA), anti-β-actin antibody (1:5000; Abcam, UK), goat anti-rabbit antibody or goat anti-mouse antibody (Beijing Zhongshan Golden Bridge BiotechnologyCo, China) were used for western blot. Trizol reagent was purchased from Invitrogen (Thermo Fisher, USA). GoScriptTM Reverse Transcription System kit was purchased from Promega (UK). SYBR Premix Ex TaqTM II was purchased from TaKaRa Clontech (Japan).

### Cell treatment

To study the effect of DMAMCL on cell survival, five RMS cell lines and NIH3T3 cell line were seeded into 96-well plates and cultured for 18 h, and then treated with DMAMCL(2.5 to 40 μM), or VCR (0.4 to 10 nM), or Epirubicin (6.25 to 500 nM) for 72 h. In combination studies of DMAMCL with VCR or Epirubicin, the two drugs were administrated at the same time. To investigate the mechanism of cell death after DMAMCL treatment, RMS cells were pretreated with Z-VAD-FMK(50 μM) (Promega, America) for 2 h before DMAMCL administration. To study the Bim expression after DMAMCL treatment, RMS cells were treated with DMAMCL (15 μM) for 0, 4, 8, 12, 16, 24 h.

### Cell survival analysis

By the end of the experiments, cell survival was evaluated using the 3-(4, 5-dimethylthiazol-2-yl)-5-(3-carboxymethoxyphenyl)-2-(4-sulfophenyl)-2H-tetrazolium, inner salt assay (MTS; inner salt assay; Promega, America) according to manufacturer’s protocol. The absorbance was measured by Microplate Reader (BIO-RAD, America) at 490 nm wavelength. The percentage of cell survival was calculated by dividing absorbance value of treated RMS cells by absorbance value of control cells within each group.

### IncuCyte live cell imaging system

Imaging was performed using the Incucyte Zoom Live-Cell Imaging System from Essen Bioscience (Ann Arbor, MI, USA). Cell confluence (%) was calculated using Incucyte Zoom software by phase-contrast images. Cells were scanned every four hours from 0 to 72 h post treatment.

### Cell cycle analysis

RMS cell lines were treated with different concentrations of DMAMCL for 48 h. All the cells (including dead cells) were collected, washed twice with phosphate-buffered saline (PBS; Bioind, Israel), fixed with cold 70% alcohol overnight, and incubated with RNase A (sigma, Amercia) at 100 μg/ml and propidium iodide (sigma, Amercia) at 50 μg/ml at room temperature away from light for 30 min. The stained cells were analyzed for DNA content by Flow cytometry (Becton, Dickinson and Company, Amercia). FlowJo software (Becton, Dickinson and Company, Amercia) was used to calculate the percentage of cells in different phases of cell cycle.

### Assay of caspase-3/7 activity

RMS cell lines were seeded into 96-well plates, cultured for 18 h, and then treated with DMAMCL (7.5 to 20 μM) for 16 h. The activity of caspase-3/7 was detected using a Caspase-Glo 3/7 Assay Kit (Promega, America) according to the manufacturer’s protocol. At the end of the drug treatment, the Caspase-Glo 3/7 reagent was added to the cells and cultured at room temperature for 1 h. Luciferase acticity was evaluated using a victor 3 luminometer (BioTec, America). DMAMCL was found to have no effect on NIH3T3 cell growth at concentrations lower than 20 μM and caspase-3/7 activity was not detected in NIH3T3 cells.

### In vivo studies

Four RMS cell lines (RD, RH18, RH30 and RH41) were used to study the effect of DMAMCL in vivo. The RMS cells were harvested, washed twice with PBS, and re-suspended in PBS and Matrigel (BD, Amercia). One hundred microliter cell suspension containing 4 × 10^6^ cells was inoculated into the right flank of 15-16 g (4–5 weeks) nude female mice (Beijing Huafukang Bioscience Co.inc., China). In the preliminary study, when the tumors reached 100–200 mm^3^, three doses of DMAMCL (50 mg/kg, 75 mg/kg and 100 mg/kg) and three doses of VCR (0.75 mg/kg, 0.85 mg/kg and 1 mg/kg) were tested individually in mice bearing RD30 xenograft tumors. Based on the body weight loss of mice and the delay of tumor growth, we selected 75 mg/kg and 100 mg/kg of DMAMCL and 0.8 mg/kg of VCR for the experiments. To study the effect of DMAMCL as a single agent, we treated the mice bearing RD, RH18, RH30 and RH41 xenograft tumors with DMAMCL (75 mg/kg, 100 mg/kg) or placebo once a day for 21 days by oral gavage. For the combination effect of DMAMCL with VCR, VCR (0.8 mg/kg) was given once on the first day of the treatment by intraperitoneal injection and DMAMCL (75 mg/kg, 100 mg/kg) was given once a day for 21 days in mice bearing RD and RH41 xenograft tumors. The tumor growth was observed for another 10 days after termination of treatment. To study the involvement of Bim in the DMAMCL treatment, mice bearing RD, RH18, RH30 and RH41 xenograft tumors were treated with 75 mg/kg DMAMCL for 4 days and tumor tissues were harvested. The expression of Bim at mRNA level or protein level was evaluated by RT-qPCR or Western Blot respectively. The tumor volumes were measured three times a week using digital caliper. The tumor volume was calculated as L*W^2^/2 (L = length, millimeter; W = width, millimeter). The mice were sacrificed when the tumors reached 200 mm^3^ for ethical reason. To study mice survival, we counted the days from the beginning of the treatment to the end of the experiment. All animal experiments were approved by the Animal Care and Use Committee of Chinese Medical University. Animals were housed at a temperature of 23 ± 2 °C and the relative humidity of 40 to 70%. All mice had free access to food and water.

### Intracellular ROS production analysis

Two RMS cell lines (RD and RH18) were used to study the effect of intracellular ROS production after DMAMCL treatment. The cells were treated with DMAMCL (10 to 20 μM) for 4 h or 6 h, and ROS generation was evaluated with a Reactive Oxygen Species Assay Kit (Beyotime, China) according to the manufacturer’s instructions.

### Protein assays

Cells samples were treated with lysis buffer (Beyotime, China) and mice xenograft tissues were treated with RIPA (Beyotime, China) and sonication. Total protein concentrations were detected using BCA or Bradford reagent (Beyotime, China). Each protein sample (30 μg) was separated on a 12% gel (Bio-Rad, Amercia), transferred to nitrocellulose membranes (Immobilon-P, Millipore, Bedford, MA, USA). The membranes were incubated in 5% milk in Tris-buffered saline with Tween20 for 2 h at room temperature to block nonspecific antibody binding, and subsequently incubated with the anti-phosphorylated-NF-κB(p65) (p-NF-κB(p65)) (1:1000), anti-Bim (1:1000), or anti-β-actin (1:5000) antibodies overnight at 4 °C. The membranes were washed with Tris-buffered saline with Tween20 and incubated with the peroxidase-conjugated goat anti-rabbit (1:5000) or anti-mouse (1:5000) antibodies for 2 h at room temperature. Bound antibodies were observed using the enhanced chemiluminescence immunoblotting detection reagent (Thermo Scientific, IL, USA).

### Quantitative RT-PCR

Total RNA was isolated from DMAMCL-treated RMS cell and tumor tissues by using Trizol reagent according to the manufacturer’s instructions. cDNA was prepared with RNA by using GoScriptTM Reverse Transcription System kit. Quantitative PCR was performed with the cDNA by using SYBR Premix Ex TaqTM II (TaKaRa Clontech) according to the manufacturer’s instructions. Beta-actin expression served as an internal control. Relative quantification of gene expression was performed with the 2(− ΔΔCt) method. Details of the PCR primers sequences were as follows: Bim sense 5′- CCCCTACCTCCCTACAGACAGA-3′, Bim anti-sense 5′- TCCAATACGCCGCAACTCTT-3′; β-actin sense 5′- AACTGGGACGACATGGAGAAA -3′, β-actin anti-sense 5′- AGGGATAGCACAGCCTGGATA -3′.

### Transfection

Bim-siRNA1, Bim-siRNA2, Bim-siRNA3 and siRNA control were purchased from Ruibo (Guangzhou, China). The sequences of siRNAs were used: Bim-siRNA1, sense: GCAACCTTCTGATGTAAGT; Bim-siRNA2, sense: CTACCTCCCTACAGACAGA; Bim-siRNA3, sense: GTATTGGAGACGATTTAA. RD and RH41cells were seed into 6 cm dishes and cultured in RPMI-1640. After 24 h culture, small interfering RNA (siRNA-Bim) or siRNA control were transfected into RD and RH41 cells using jetPRIME agent (Polyplus Transfection, Illkirsch, France) according to the manufacturer’s instructions. Sixteen hours after transfection, RMS cells were harvested and seeded into 96-plate well and 6-well plate, and then treated with DMAMCL. After 16 h of DMAMCL treatment, cells in 6-well plates were harvested and proteins were extracted from these cells for Bim expression study. Cells in 96-well plates were used for cell survival analysis by MTS assay after 72 h of DMAMCL treatment.

### Statistical analysis

Results were shown as means ± standard deviation (SD) for in vitro cellular experiments and means ± standard error (SE) for in vivo murine experiments. The synergistic effects between two agents in in vitro experiments were evaluated by combination index (CI) with the CompuSyn software. Comparisons between 2 groups were performed using Student’s t test. Survival analysis between multiple groups were done using Log-Rank test. The survival curve was drawn using GraphPad Prism 7 software. Statistical significance was determined as *P* < 0.05 (*) or *P* < 0.001 (**).

## Results

### DMAMCL treatment induces cell death in RMS cells in vitro

To study the effect of DMAMCL on RMS cell survival in vitro, RD, RH18, and RH28, RH30, RH41 were treated with different concentrations of DMAMCL for 72 h, at the end of the experiments MTS assay was used to detect the cell survival. Cell confluency (% of the surface area of cells) was dynamically detected and calculated using Incucyte Zoom software based on phase-contrast images.

**Fig. 1:**
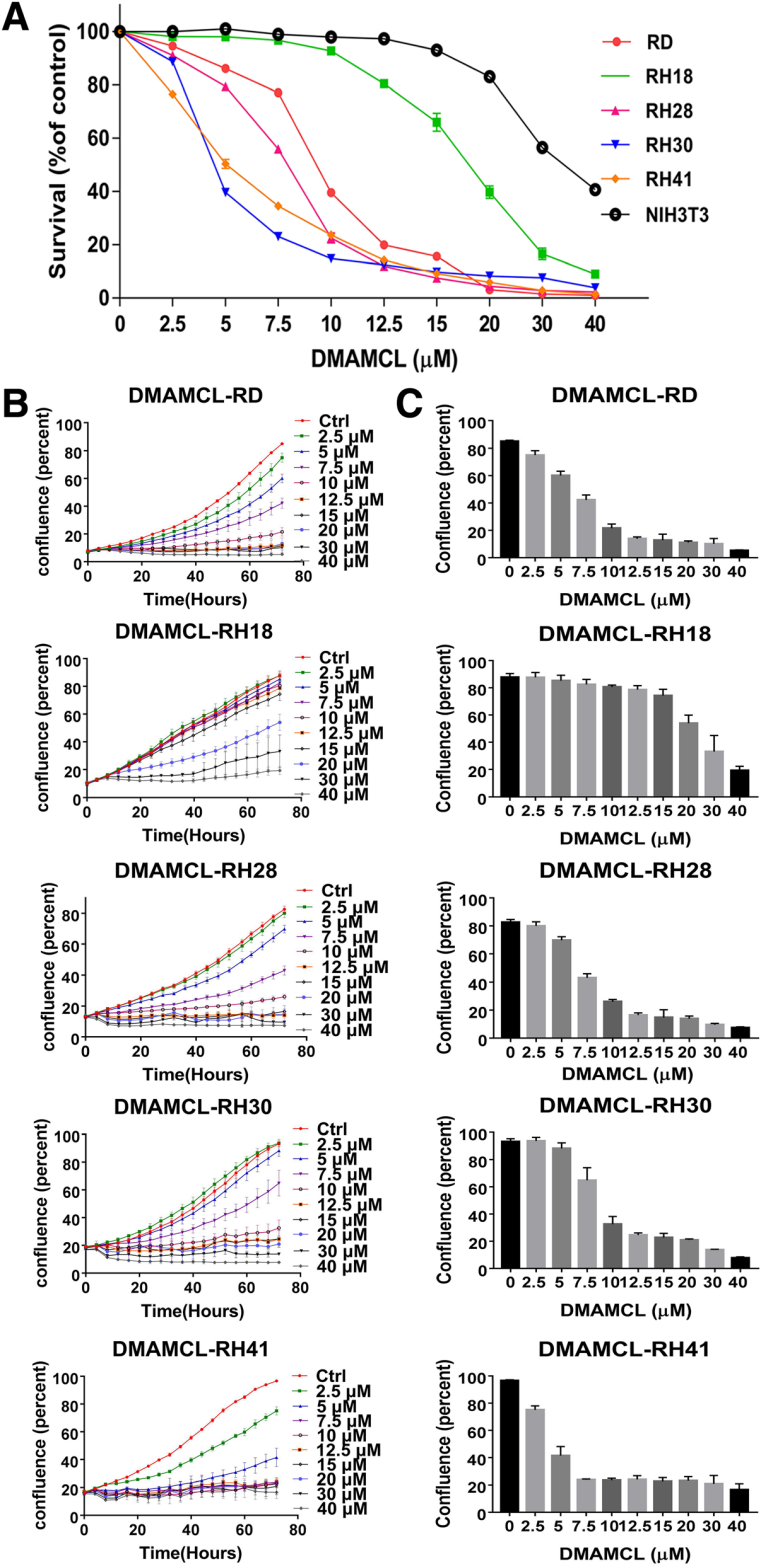
DMAMCL treatment induces cell death in RMS cells in vitro. **a** Rhabdomyosarcoma(RMS) cell lines and control NIH3T3 cells were treated with different concentrations of DMAMCL for 72 h. Cell survival was evaluated by MTS. Each data point represents the mean, SD of triplicate wells. **b** Cell confluency(%) was calculated using Incucyte Zoom software based on phase-contrast images of RD, RH18, RH28, RH30 and RH41 cells from 0 h to 72 h at different concentration of DMAMCL. Each data point represents triplicate wells. **c** Cell confluency(%) was calculated using Incucyte Zoom software based on phase-contrast images of RD, RH18, RH28, RH30 and RH41 cells at 72 h. Each data point represents triplicate wells

DMAMCL treatment induced a concentration-dependent cell death in these RMS cells evaluated using MTS assay. The IC50s of RH30, RH41, RH28, RD and RH18 were 4.68 μM, 4.96 μM, 7.03 μM, 8.73 μM and 22.16 μM respectively; while the IC50 of a mouse fibroblast cell line NIH3T3 was 34.63 μM, indicating that normal cells are more resistant to DMAMCL treatment (Fig. 1). DMAMCL dose effect in different RMS cell lines was further measured by cell confluence using IncuCyte in real time, and similar dose responses were observed (Fig. 1b and c). These results suggest that DMAMCL treatment suppressed cell proliferation or induced dose-dependent cell death in RMS cells.

### DMAMCL induced RMS cell death via apoptosis in vitro

**Fig. 2:**
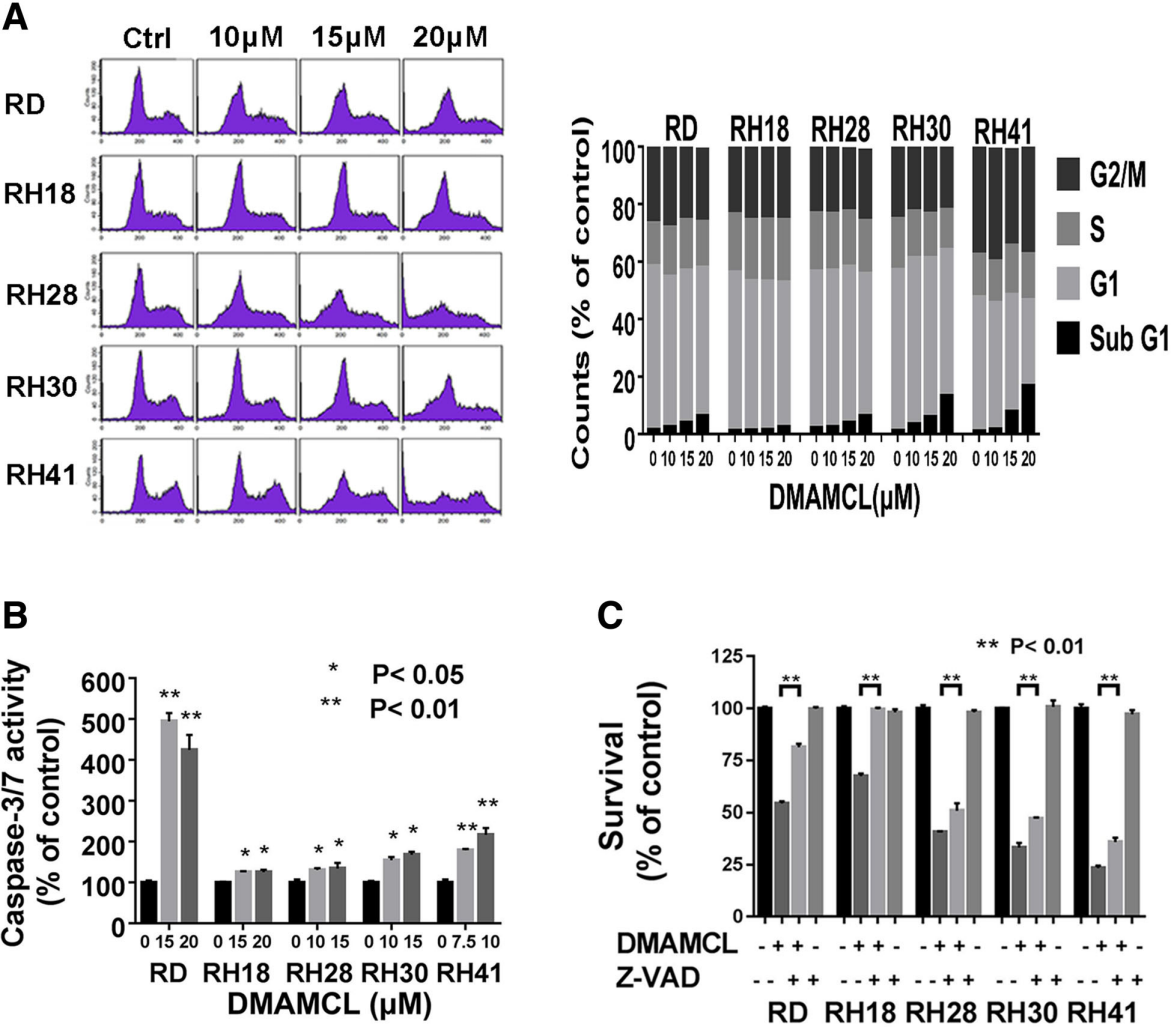
DMAMCL induces RMS cell death via apoptosis in vitro. **a** RD, RH18, RH28, RH30 and RH41 cells were treated with different concentrations of DMAMCL for 48 h, harvested the cells with dead cells stained with propidium iodide. The percentage of cells in Sub-G1, G1 and S-G2–M phases is shown. **b** RD, RH18, RH28, RH30 and RH41 cells were treated with different concentrations of DMAMCL for 16 h, caspase-3/7 activity was evaluated. Each data point represents the mean, SD of triplicate wells. **c** RD, RH18, RH28, RH30 and RH41 cells were pretreated with Z-VAD-FMK (50 μM) for 2 h followed by the treatment of different concentrations of DMAMCL (RD for 10 μM, RH18 for 15 μM, and RH28, RH30 and RH41 for 7.5 μM). Cell survival was evaluated 48 h later by MTS. Each data point represents the mean, SD of triplicate wells

To investigate whether DMAMCL represses RMS cell cycle progression or induces cell death via apoptosis, we treated RMS cells with different concentrations of DMAMCL (10 μM, 15 μM, 20 μM) for 48 h, harvested all the cells and performed cell cycle analysis using a flow cytometer. Figure 2a (left) showed that there was a concentration-dependent increase of cells in the SubG1 phase in the cell cycle after DMAMCL treatment in RD, RH18, RH28, RH30 and RH41 cell lines, but no G1 or G2-M arrest was observed. The increases of cells in SubG1 phase at 20 μM were 6.33% in RD cells, 2.47% in RH18 cells, 6.28% in RH28 cells, 13.26% in RH30 cells, and 16.76% in RH41 cells respectively (Fig. 2a, right). We examined the activity of caspase 3/7 after DMAMCL treatment (16 h). Figure 2b showed that caspase 3/7 activity increased after DMAMCL treatment in all the 5 RMS cell lines, from 125% of control in RH18 cells to 495% of control in RD cells. To study whether the DMAMCL-induced cell death was caspase-dependent, we pretreated the RMS cells with the pan-caspase inhibitor Z-VAD-FMK (50 μM) for 2 h, and then treated with DMAMCL (RD for 10 μM; RH18 for 15 μM; RH28, RH30, RH41 for 7.5 μM) for 48 h. The MTS assay was used to detect the cell survival. Figure 2c showed that pretreatment with Z-VAD-FMK blocked the DMAMCL-induced cell death in all the five tested RMS cell lines. These data indicated that DMAMCL treatment led to a caspase-dependent apoptosis in RMS cells.

### The combination effect of DMAMCL with VCR or Epirubicin in RMS cells in vitro

**Fig. 3:**
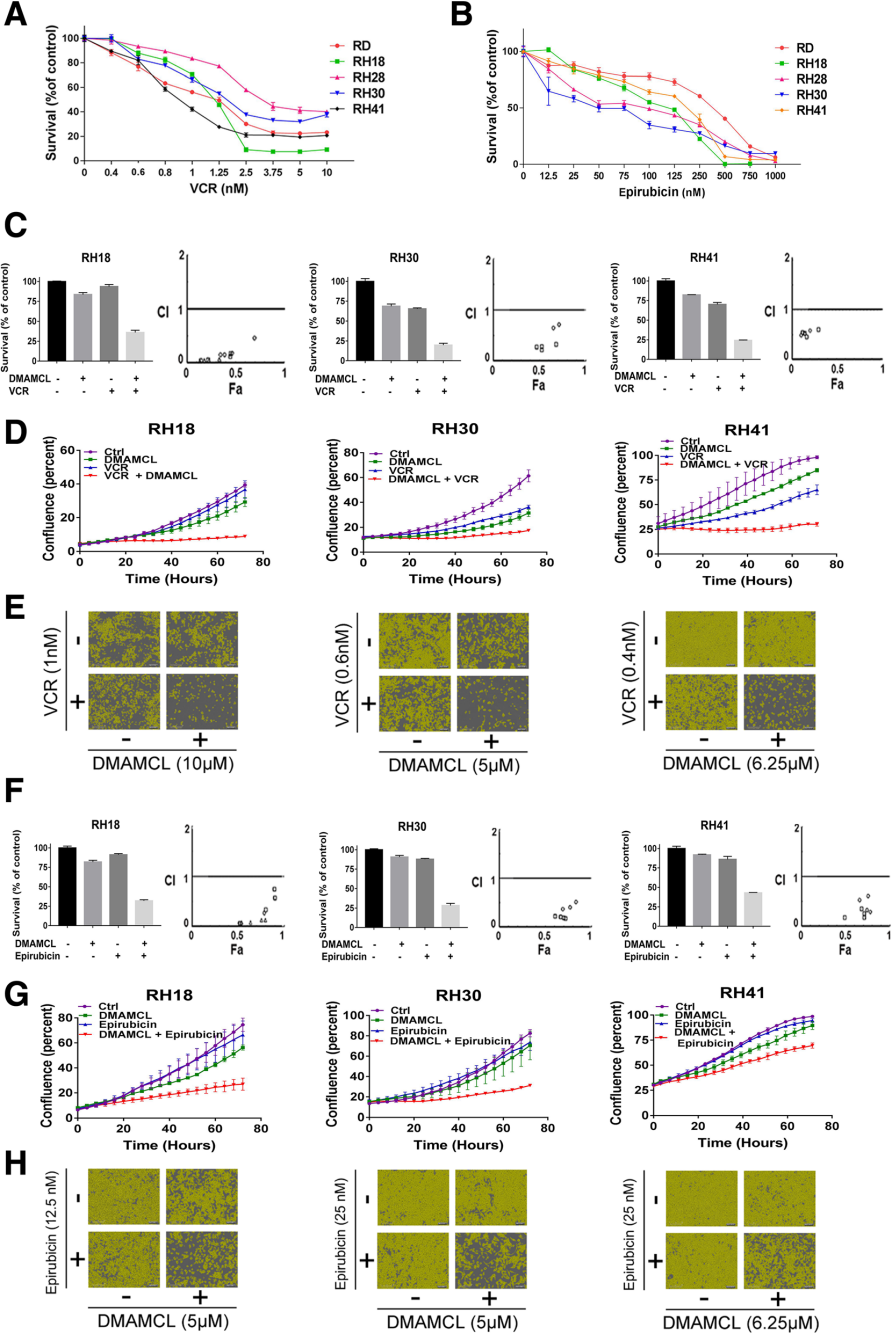
The combination effect of DMAMCL with VCR or Epirubicin in RMS cells in vitro. **a** RMS cell lines were treated with VCR for 72 h. Cell survival was evaluated by MTS. Each data point represents the mean, SD of triplicate wells. **b** RMS cell lines were treated with Epirubicin for 72 h. Cell survival was evaluated by MTS. Each data point represents the mean, SD of triplicate wells. **c** RH18, RH30 and RH41 cells were treated with DMAMCL and VCR at different concentration in combination for 72 h. Cell survival was evaluated by MTS. Each data point represents the mean, SD of triplicate wells. The combination study was value by CI. CI < 1 indicates synergism, CI = 1 reflects an additive effect, and CI > 1 indicates drug antagonism. **d** RH18, RH30 and RH41 cells were treated with DMAMCL and VCR at different concentration in combination from 0 h to 72 h. Cell confluency(%) was calculated using Incucyte Zoom software by phase-contrast images. Each data point represents triplicate wells. **e** The pictures of RH18, RH30 and RH41 cells were treated with DMAMCL and VCR either alone or in combination for 72 h. **f** RH18, RH30 and RH41 cells were treated with DMAMCL and Epirubicin at different concentration in combination for 72 h. Cell survival was evaluated by MTS. Each data point represents the mean, SD of triplicate wells. The combination study was value by CI. **g** RH18, RH30 and RH41 cells were treated with DMAMCL and Epirubicin at different concentration in combination from 0 h to 72 h. Cell confluency(%) was calculated using Incucyte Zoom software by phase-contrast images. Each data point represents triplicate wells. **h** The pictures of RH18, RH30 and RH41 cells were treated with DMAMCL and Epirubicin either alone or in combination for 72 h

VCR and Epirubicin are chemotherapeutic agents used in the clinical treatment of patients with RMS who have considerable side effects when given at high doses. To investigate the combination effect of DMAMCL with VCR or Epirubicin, we first studied the effect of VCR or Epirubicin as a single agent on RMS cells. Figure 3a and b showed that VCR or Epirubicin induced a concentration-dependent cell death in the five RMS cell lines (RD, RH18, RH28, RH30 and RH41) which were detected using MTS assay after 72 h of treatment. Additional file 1: Figure S1A and S1B showed that cell confluence changed as the time extension from 0 to 72 h at different concentrations of VCR or Epirubicin. Based on these results, we further studied the combination effect of DMAMCL with VCR or Epirubicin. The five RMS cell lines were treated with DMAMCL and chemotherapeutic drugs (VCR or Epirubicin) for 72 h, individually or in combination. The cell survival or cell confluency was detected by MTS assay (Fig. 3c, f, Additional file 2: Figure S2 A, D) or Incucyte machine (Fig. 3d, g, Additional file 2: Figure S2 B,E) respectively. Figure 3c (left of each cell line) showed that the cell survival in the cells treated with a combination of DMAMCL and VCR was much lower than those in the cells treated with each individual agent (combination vs DMAMCL or VCR: 36% vs 83% or 93% in RH18 cells, 20% vs 69% or 65% in RH30 cells, 25% vs 86% or 67% in RH41). Similar results were found in RD and RH28 cells (Additional file 2: Figure S2A). Selected combinations of DMAMCL and VCR or Epirubicin were examined for each cell line and the synergistic effect was evaluated by calculating a CI value using ComboSyn software. A CI < 1 indicates synergism and CI > 1 indicates drug antagonism. A synergistic effect between DMAMCL and VCR was observed in the 5 RMS cell lines as the CI values were smaller than 1 (Fig. 3c (right of each cell line) and Additional file 2: Figure S2 A-lower lane). Figure 3f (left of each cell line) showed that the cell survivals in the cells treated with a combination of DMAMCL and Epirubicin were much lower than those in the cells treated with each individual agent (combination vs DMAMCL or Epirubicin: 32% vs 82% or 91% in RH18 cells, 28% vs 91% or 88% in RH30 cells, 43% vs 92% or 86% in RH41). Similar results were found in RD and RH28 cells (Additional file 2: Figure S2D). A synergistic effect between DMAMCL and Epirubicin was observed in the 5 RMS cell lines as the CI values were smaller than 1 (Fig. 3f (right of each cell line) and Additional file 2: Figure S2 D-lower lane). Figure 3d and g showed the dynamic changes of cell confluency after DMAMCL and/or VCR/Epirubicin treatment in RH18, RH30 and RH41 cells. The cell confluence of cells treated with a combination of DMAMCL and VCR (Fig. 3d) / Epirubicin (Fig. 3g) was significantly lower than those of cells treated with each individual. Similar results were found RD and RH28 cells (Additional file 2: Figure S2 B,E). Figure 3e, h and Additional file 2: Figure S2 C,F were the representative changes of RH18, RH30, RH41 and RD,RH28 cells under microscope after a combination treatment of DMAMCL with VCR or Epirubicin. The number of cells was significantly decreased in the combination groups compared to each individual agent groups. These data indicated that a combination of DMAMCL with chemotherapeutic drugs significantly increased cell death compared to single agent in RMS cells.

### The effect of DMAMCL as a single agent on tumor growth and mice survival in vivo

**Fig. 4:**
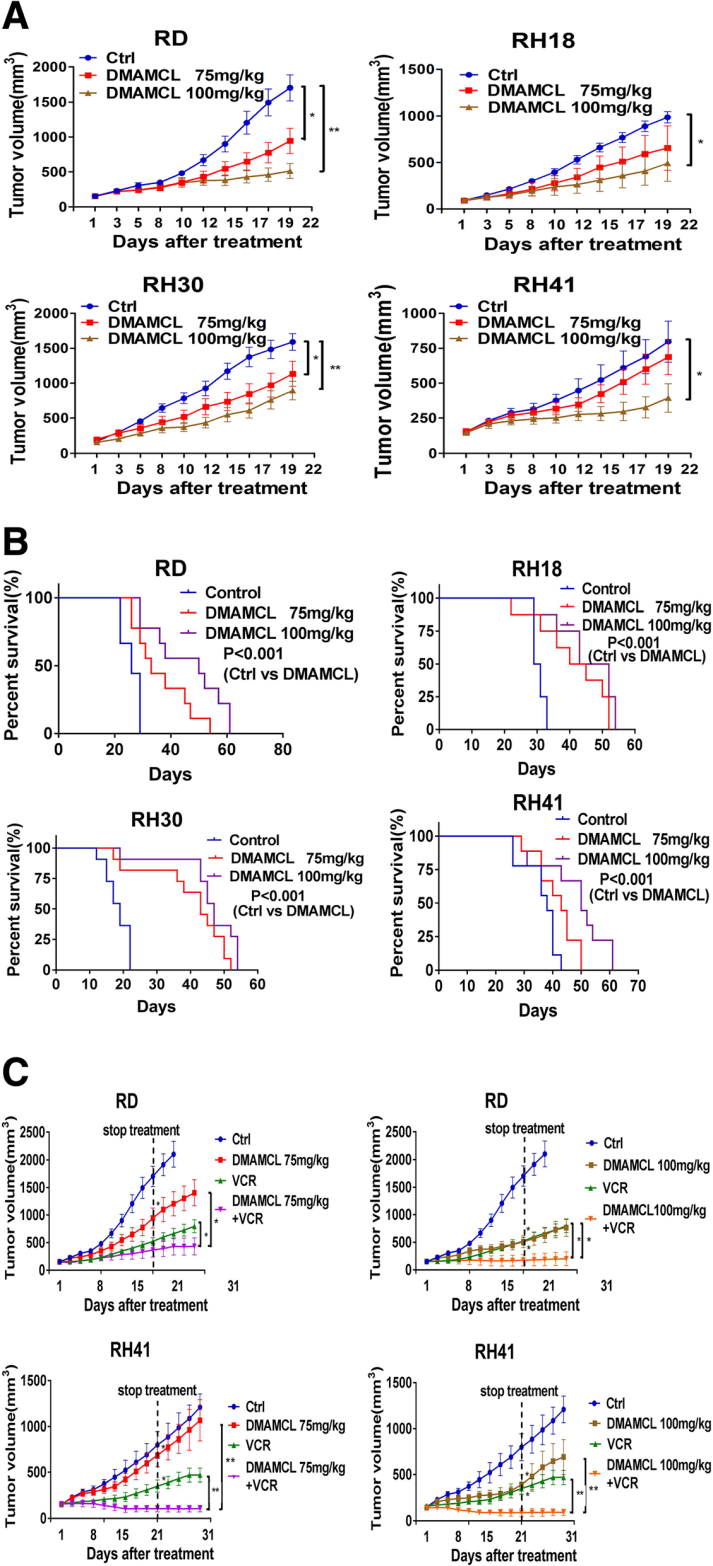
The effect of DMAMCL as a single agent or in combination with VCR on tumor-growth and mice-survival. RD, RH18, RH30, and RH41 cells were injected into mice. Mice with tumors were treated with vehicle, DMAMCL (75 or 100 mg/kg/d), VCR(0.8 mg/kg) or DMAMCL (75 or 100 mg/kg/d) with VCR(0.8 mg/kg) for 21 days. The dimension of the tumor volume was measured three times a week by digital caliper. The tumor volume was calculated as L*W^2^/2 (L = length, millimeter; W = width, millimeter). **a** Tumor volumes from control and treatment groups were compared when the last mouse in each group was euthanized. Bar, SE, *, *P* < 0.05; **, *P* < 0.001, RD (*n* = 9), RH18 (*n* = 8), RH30 (*n* = 11), and RH41 (*n* = 9). **b** Survival curve was plotted by Kaplan–Meier analysis. *, *P* < 0.05; **, *P* < 0.001, treatment groups versus control group, RD (*n* = 9), RH18 (*n* = 8), RH30 (*n* = 11), and RH41 (*n* = 9). **c** Tumor volumes from control and treatment groups were compared when the last mouse in each group was euthanized. Bar, SE, *, *P* < 0.05; **, *P* < 0.001, RD (*n* = 9), RH18 (*n* = 8), RH30 (*n* = 11), and RH41 (*n* = 9)

To evaluate whether DMAMCL had any effect on RMS tumor growth in vivo, we treated the mice bearing RD, RH18, RH30 and RH41 xenograft tumors with DMAMCL at 75 mg/kg/day or 100 mg/kg/day for 21 days. Figure 4a showed that at the end of treatment, compared to the control group, the DMAMCL-induced inhibition of tumor growth at 75 mg/kg/day dose was 69.79% in RD tumors (*P* < 0.05), 33.58% in RH18 tumors (*P* = 0.10), 28.70% in RH30 tumors (*P* < 0.05) and 13.84% in RH41 tumors (*P* = 0.29); and at 100 mg/kg/day dose the DMAMCL-induced inhibition of tumor growth was 69.79% in RD tumors (*P* < 0.001), 50.1% in RH18 tumors (*P* < 0.05), 43.77% in RH30 tumors (*P* < 0.001) and 50.55% in RH41 tumors (*P* < 0.05). Although DMAMCL treatment at the dose of 75 mg/kg/day didn’t induce statistical inhibition of tumor growth in mice bearing RH18 and RH41 tumors, there was still a trend toward tumor growth inhibition.

The mice survival after DMAMCL treatment was also studied and significant survival advantage in DMAMCL-treated mice bearing RMS xenograft tumors was observed. Figure 4b showed that the median survival time for control versus DMAMCL 75 mg/kg/day and 100 mg/kg/day was 26 days versus 33 days (*P* < 0.001) and 50 days (*P* < 0.001) in mice bearing RD tumors, 30 days versus 42.5 days (*P* < 0.001) and 47.5 days (*P* < 0.001) in mice bearing RH18 tumors, 19 days versus 43 days (*P* < 0.001) and 47 days (*P* < 0.001) in mice bearing RH30 tumors, 38 days versus 43 days (*P* < 0.001) and 50 days (*P* < 0.001) in mice bearing RH41 tumors. There was no significant change in the weight of mice bearing RMS tumors during DMAMCL treatment (Additional file 3: Figure S3). These data indicated that as a single agent DMAMCL had anti-tumor growth effect and increased the survival of mice bearing RMS xenograft tumors.

### The combination effect of DMAMCL with VCR on tumor growth in vivo

To assess the combination effect of DMAMCL with VCR in vivo, we treated the mice bearing RD and RH41 tumors with DMAMCL (75 mg/kg, or 100 mg/kg) or VCR (0.8 mg/kg) alone, or in combination for 21 days, and then kept observing the changes of tumor sizes for another 10 days. At day 21 of treatment, the combination of DMAMCL (100 mg/kg/day) and VCR, but not either single agent alone, completely inhibited the tumor growth, showing statistically higher inhibition of tumor growth compared to each individual agent in both RD and RH41 tumors (Fig. 4c, right). Even we stopped the treatment at day 21 and kept observing the tumor growth until day 31, the tumors in mice treated with the combination of two drugs failed to grow (Fig. 4c, right). A combination of DMAMCL at 75 mg/kg dose and VCR also decreased tumor growth compared to each individual agent in RD and RH41 tumors, although the inhibition of tumor growth reached statistical difference until day 31 (not at day 21) in RD tumors (Fig. 4c, left). These data indicated that the combination of DMAMCL with VCR significantly increased the anti-tumor growth effect compare to each individual agent in mice bearing RMS xenograft tumors.

### Bim partially mediates the DMAMCL-induced cell death in RMS cells

**Fig. 5:**
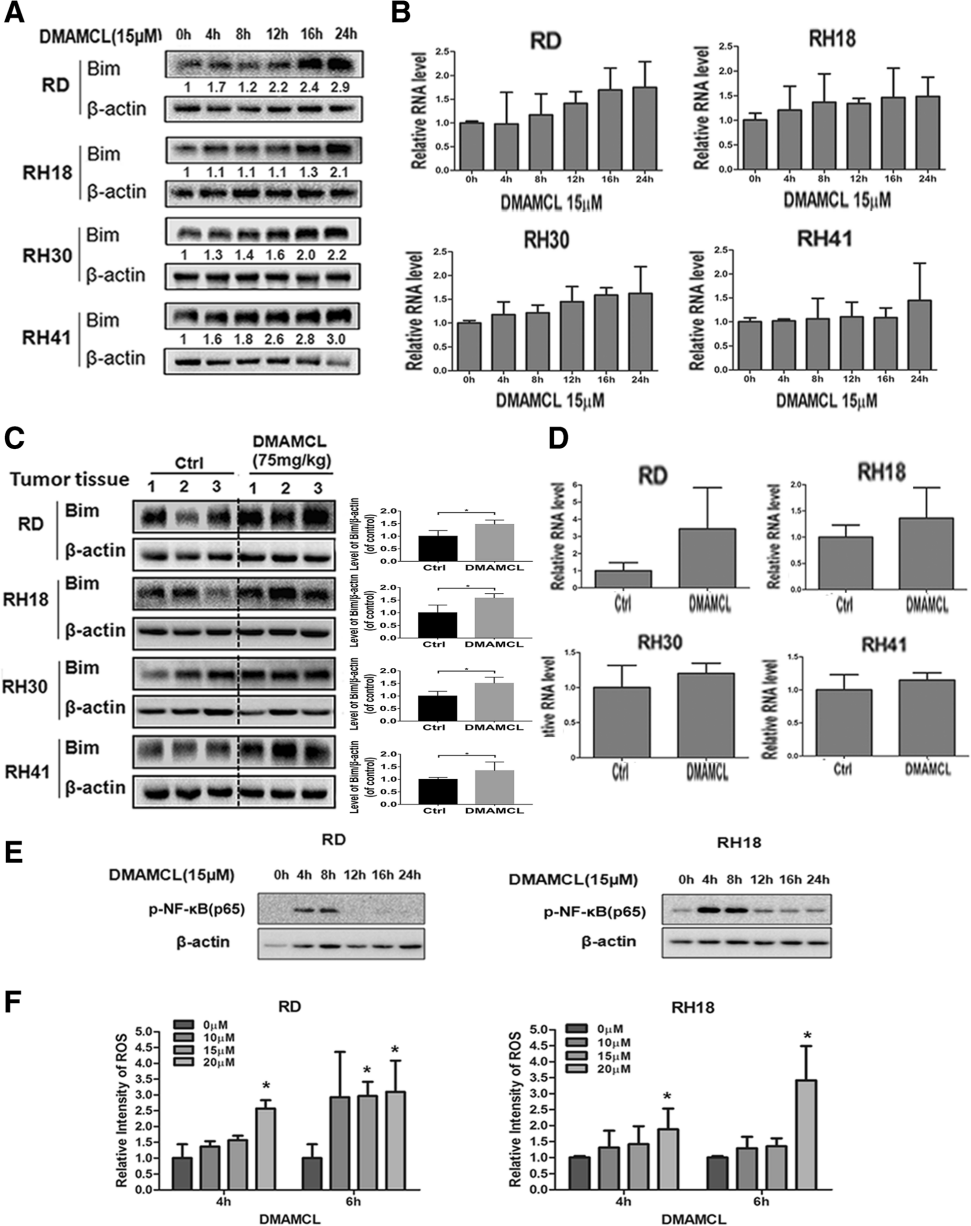
The changes of Bim expression, p-NF-κB(p65) expression and ROS genenration after DMAMCL treatment in RMS cells. RD, RH18, RH30, and RH41 cells were treated with DMAMCL for 0, 4, 8, 12, 16, 24 h, total protein and total RNA was extracted for analysis of anti-Bim and anti-β-actin by western blot (**a**) and RT-qPCR (**b**). RMS xenografte-tumors were harvested at end points, total protein and total RNA was extracted for analysis of anti-Bim and anti-β-actin by western blot (**c**) and RT-qPCR (**d**). **e** RD and RH18 cells were treated with DMAMCL for 0, 4, 8, 12, 16, 24 h, total protein was extracted for analysis of anti-p-NF-κB(p65) and anti-β-actin by western blot. **f** RD and RH18 cells were treated with DMAMCL for 0, 4, 6 h, and the activity of ROS was detected. Bar, SD, *, *P* < 0.05

To study the potential mechanism of DMAMCL-induced cell death, we screened the changes of BCL-2 family members after DMAMCL treatment, and found that there was an increase of Bim expression in all the tested 4 RMS cell lines (RD, RH18, RH30 and RH41) in vitro and in vivo (Fig. 5a, b, c and d). RMS cells were treated with 15 μM of DMAMCL, and harvested at different time points (0, 4, 8, 12, 16, 24 h). An increased expression of Bim began to be detected at 4 h in RD, RH30 and RH41 cells, or at 16 h in RH18 cells, and continued to increase up to 24 h at protein level (Fig. 5a). There was a trend of increased expression of Bim mRNA in the 4 RMS cell lines (RD, RH18, RH30 and RH41) which was not statistically significant (Fig. 5b). We also treated the mice bearing RMS xenograft tumors with DMAMCL (75 mg/kg) for 4 days, harvested the tumor tissues to evaluate the Bim expression. The expression of Bim in DMAMCL treated-RMS tumors was higher than that in the untreated RMS tumors (Fig. 5c) at protein level and the densitometry analysis was shown in Fig. 5c (right). RT-qPCR results showed that the expression of Bim had an increased trend at RNA level in DMAMCL treated-RMS tumors compared to the untreated RMS tumors which was not statistically significant (Fig. 5d).

Since NF-κB and the production of ROS have been reported to mediate the effect of DMAMCL and MCL in some cancers [28, 29], we next evaluated the activity of NF-κB and ROS production in RMS cells before and after DMAMCL treatment. For the NF-κB activity, we detected the expression changes of phosphorylated-NF-κB(p65). p65 is a subunit of NF-κB and phosphorylation of p65 has been reported to induce cell apoptosis [31, 32]. We treated the RMS cells (RD and RH18) with 15 μM DMAMCL for 0, 4, 8, 12, 16, 24 h and found that the expression of p-NF-κB (p65) had a transient increase (at 4 and 8 h) and began to decrease at 12 to 24 h in both RD and RH18 cells (Fig. 5e). The activity of ROS in RD and RH18 cells was detected after the cells were treated with DMAMCL (0, 10, 15, and 20 μM) for 4 h and 6 h. Our results showed that there were significant increases in ROS activity in DMAMCL-treated RD and RH18 cells, and there was a statistically significant difference at dose of 20 μM (*P* < 0.05) (Fig. 5f). These data suggested that Bim, NF-κB and ROS may all contribute to the effects of DMAMCL in RMS cells.

To further investigate whether Bim is mediated the DMAMCL-induced cell death in RMS cells, we down-regulated the Bim expression by transfecting Bim siRNA into the RD and RH41 cells, and then treated the cells with DMAMCL. Cell survival was detected by MTS assay and cell confluence was measured by Incucyte live cell imaging system.

**Fig. 6:**
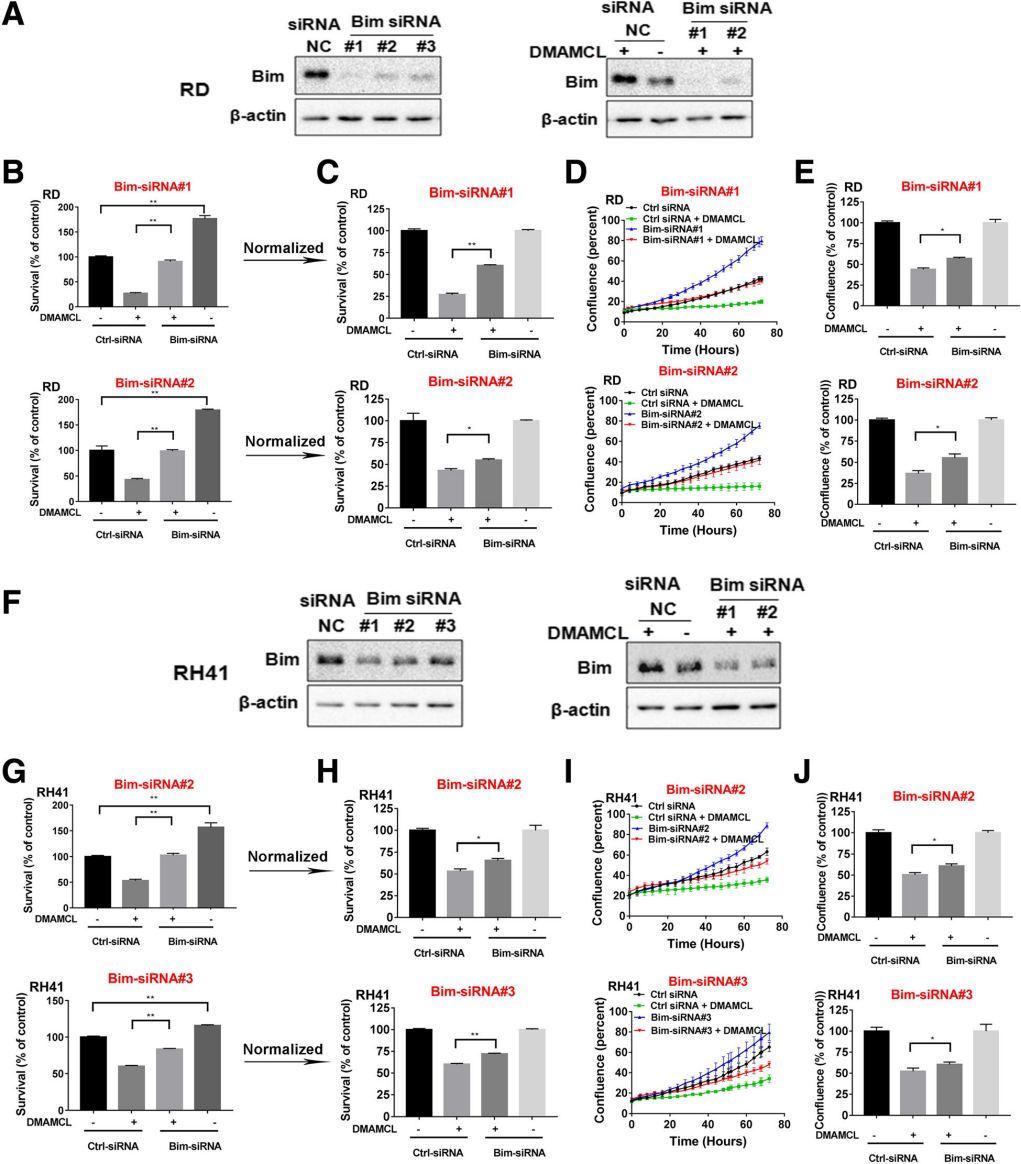
Bim partially mediates the DMAMCL-induced cell death in RMS cells. RD cells were transfected with Bim-siRNAs and control-siRNA. 16 h later, the cells were treated with DMAMCL for 72 h. **a** RD cells were transfected with Bim-siRNAs and control-siRNA with or without DMAMCL by western blot. **b** Cell survival was detected by MTS assay. Each data point represents triplicate wells. **c** Cell survival was detected by MTS assay after normalized. Each data point represents triplicate wells. **d** Cell confluency(%) was calculated by Incucyte Zoom software by phase-contrast images from 0 to 72 h. Each data point represents triplicate wells. **e** Cell confluency(%) was calculated by Incucyte Zoom software by phase-contrast images from 0 to 72 h after normalized. Each data point represents triplicate wells. RH41 cells were transfected with Bim-siRNAs and control-siRNA. 16 h later, the cells were treated with DMAMCL for 72 h. **f** RH41 cells were transfected with Bim-siRNAs and control-siRNA with or without DMAMCL by western blot. **g** Cell survival was detected by MTS assay. Each data point represents triplicate wells. **h** Cell survival was detected by MTS assay after normalized. Each data point represents triplicate wells. **i** Cell confluency(%) was calculated by Incucyte Zoom software by phase-contrast images from 0 to 72 h. Each data point represents triplicate wells. **j** Cell confluency(%) was calculated by Incucyte Zoom software by phase-contrast images from 0 to 72 h after normalized. Each data point represents triplicate wells

Three Bim siRNAs (#1, #2, #3) were designed and all of them down-regulated the endogenous Bim expression at protein level in RD and RH41 cells (Fig. 6a and f, left). We selected Bim siRNA (#1, #2 for RD and #2, #3 for RH41) for the following study. The DMAMCL-induced increase of Bim was blocked by transfection of Bim siRNA into RD and RH41 cells (Fig. 6a and f, right). We found that the down-regulation of Bim expression in RMS cells increased the cell survival detected by MTS assay in the absence DMAMCL treatment. Figure 6b and g showed that the survival of cells transfected with Bim siRNA was much higher than that of cells transfected with control siRNA (% of control, Bim siRNA #1: 176.76% in RD cells, Bim siRNA #2: 179.13% in RD cells, Bim siRNA #2: 156.5% in RH41 cells; Bim siRNA #3: 115.75% in RH41 cells). To evaluate the effect of Bim down-regulation on DMAMCL-induced cell death, we normalized the survival of cells treated with DMAMCL by the survival of untreated cells. We found that, for the cells treated with DMAMCL, the survival of cells transfected with Bim siRNA (#1or #2) was statistically higher than the survival of cells transfected with control siRNA: Bim siRNA #1 versus control siRNA: 60.12% versus 26.86% in RD cells, Bim siRNA #2 versus control siRNA: 55% versus 43.09% in RD cells, Bim siRNA #2 versus control siRNA: 65.62% versus 53.14% in RH41 cells; Bim siRNA #3 versus control siRNA: 72.17% versus 60.12% in RH41 cells)(Fig. 6c for RD cells and 6H for RH41 cells).

Next we investigated the effect of Bim in DMAMCL treated cells by detecting the confluency of cells using Incucyte Zoom live cell imaging system. Figure 6d and i showed the dynamic changes of cell confluency under different conditions. The confluency curve of Bim siRNA-transfected cells (blue curve) was higher than that of the control siRNA-transfected cells (black curve), indicating the more proliferation in Bim siRNA-transfected cells. This result was similar to the survival changes detected by MTS assay in Fig. 6b and g.

To study the effect of Bim down-regulation on DMAMCL-induced cell death, we used the cell confluency data at 72 h of treatment and normalized the confluency of DMAMCL-treated, Bim siRNA-transfected cells by that of the untreated Bim siRNA-transfected cells as in Fig. 6e and j. We found that, for the cells treated with DMAMCL, the proliferation of cells transfected with Bim siRNA (#1, #2 or #2, #3) was statistically higher than the proliferation of cells transfected with control siRNA: Bim siRNA #1 versus control siRNA: 56.95% versus 43.89% in RD cells, Bim siRNA #2 versus control siRNA: 55.39% versus 36.77% in RD cells; Bim siRNA #2 versus control siRNA: 60.66% versus 50.32% in RH41 cells, Bim siRNA #3 versus control siRNA: 60.34% versus 52.11% in RH41 cells)(Fig. 6e for RD cells and 6 J for RH41 cells). These data indicated that down-regulation of Bim expression partially blocked the DMAMCL-induced cell death.

## Discussion

Radiotherapy and chemotherapy kill a large number of tumor cells by the damage of DNA, inhibition of mitosis and induction of apoptosis, but they often miss the slow dividing and non-dividing cells (G0 stage cells and cancer stem cells, CSC). These cancer cells often lead to cancer recurrence, metastasis, drug resistance, which are still the main causes of death of RMS [33]. Furthermore, lack of improvement with conventional chemotherapy and target therapy has highlighted the need for new and effective treatments or new agents. In the present study, we found that the novel agent DMAMCL had anti-tumor growth effect in vitro and in vivo in RMS as a single agent and a combination of DMAMCL with chemotherapeutic drugs (Vincristine, or Epirubicin) achieved a significantly increased anti-tumor effect compared to the individual agent alone. DMAMCL treatment of RMS up-regulated Bim protein level and ROS production, transiently increased p-NF-κB(p65) protein level. Knockdown of Bim partially blocked DMAMCL-induced cell death, indicating that Bim may partially mediate the DMAMCL-induced caspase-dependent apoptosis in RMS cells.

Parthenolide (PTL), Micheliolide (MCL) and DMAMCL belong to sesquiterpene lactone family (SLs), which are isolated from traditional Chinese medicine. PTL is known as anti-tumor and anti-inflammation molecules from Asteraceae family, but it is unstable in both acidic and basic conditions [18–20, 34]. MCL is more stable than PTL in vivo [22]. Before the development of DMAMCL, the effect of MCL has been studied in many types of cancers. MCL reduces the proportion of acute myelogenous leukemia stem cells (AML stem and progenitor cells) in vitro, and inhibits the tumor volumes in nonobese diabetic/severe combined immunodeficiency AML models in vivo by oral [22, 35]. MCL inhibits the LPS-induced intestinal inflammation and colitis-associated cancer in vitro and in vivo [28], induces the cells death of breast cancer cells in vitro [29], strengthens the cisplatin sensitivity in breast cancer cells in vitro and in vivo [30]. But MCL is unstable at room temperature.

DMAMCL, a pro-drug of MCL, continuously releases MCL in plasma for 8 h and is more stable, more active, and less toxic to normal cells and normal stem cells. DMAMCL is a novel agent that has an effect of anti-tumor growth and anti-inflammation. DMAMCL reduces tumor proliferation in glioma cells in vitro and in vivo [23], and selectively deracinates AML stem and progenitor cells in vitro and in vivo [36]. DMAMCL significantly suppresses the growth of leukemia cells in vitro and tumorigenesis in a zebrafish xenograft model in vivo [35]. These reports showed that DMAMCL or MCL as a single anti-tumor agent has an effect of anti-tumor or anti-cancer stem cells growth in adult cancers [23, 28–30, 36–38], but to date there have been no reports on its effect in children tumors. In the present study, we compared the sensitivity to DMAMCL between RMS cells and NIH3T3 cells (a mouse fibroblast cell line), and found that the IC50 in RMS cells ranged from 4.68 to 22.16 μM, while the IC50 of NIH3T3 cells was 34.64 μM which was 1.56 to 7.4 fold of those in RMS cells. These results indicated that DMAMCL had less effect on normal cells compared to RMS cells, and DMAMCL is an anti-tumor agent in RMS.

Decreasing cytotoxicity and increasing the treatment efficacy are the ideal goals for tumor treatment, thus identifying rational combinations of different agents is a common strategy for cancer therapy [39]. In a combination study, MCL strengthenes the cisplatin sensitivity in MCF-7 cells of breast cancer in vitro and in vivo. The authors first treat the MCF-7 cells with cisplatin for 24 h to increase the number of cancer stem cells, then treat the cells with MCL [30]. In our study, we added DMAMCL with VCR or Epirubicin at the same time, we found a significant combination effect in RMS cells in vitro and in vivo. To our knowledge, the evaluation of DMAMCL effect on pediatric tumors in combination with either chemotherapeutic drugs was not reported before. Our data will provide support for clinical trials in RMS patients and for evaluation of DMAMCL in other pediatric tumors.

The mechanisms underlying anti-tumor cell growth effect of MCL or DMAMCL have been investigated. MCL inhibits colitis-associated cancer cell proliferation via activating the NF-κB pathway [28]. MCL induces the cell death of breast cancer cells by increasing mitochondrial fission, decreasing mitochondrial membrane potential, releasing reactive oxygen species (ROS) generation, disrupting PARP and up-regulating Drp1 expression [29]; MCL enhances the cisplatin sensitivity in MCF-7cells of breast cancer in vitro and in vivo via increasing the expression of ALDH+ (the marker of breast cancer stem cell) and up-regulating Krüppel-like factor 4 (KLF4, cell self-renewal and maintenance of pluripotency) expression [30]. DMAMCL reduces tumor proliferation via down-regulating anti-apoptosis gene Bcl2 and increasing apoptosis in a dose-dependent manner in glioma cells in vitro and in vivo [23]. DMAMCL selectively reduces AML stem and progenitor cells by inhibiting NF-κB activity and increasing intracellular reactive oxygen species (ROS) generation [22, 36]. Consistently, here we found that DMAMCL treatment induced an transient increase of p-NF-κB(p65) and an increase of ROS production in RMS cells. Although it has never been reported, in our study, we found pro-apoptosis gene Bim increased after DMAMCL treatment in RMS cells and partially mediated the DMAMCL effect. Bim as a BH3-only protein is important on initiating the intrinsic apoptosis pathway both in physiological and pathophysiological conditions [40, 41]. Bim is essential for immune response, which induces apoptosis by stimulation of a large number of growth factors or cytokine deletion, calcium flux, banding antigen receptors on T and B cells, loss of adhesion, microtubule disruption and tyrosine kinase inhibitors. The absence of Bim could promote autoimmunity dysfunction, chronic inflammation, tumor progression, drug-resistance and therapy failure [42–46]. Recently, it is reported that ONC201 could induce apoptosis by increasing the levels of Bim in myeloma cells [47], AZD9291 (the third generation EGFR inhibitor) could increase the expression of Bim by MEK/ERK-dependent pathway in EGFR-mutant non-small cell lung cancer (NSCLC) cells [48], and the combination of pimasertib (MEK inhibitor) with SAR405838 (MDM2 inhibitor) could induce the expression of Bim in vitro and in vivo in KRAS mutant non-small cell lung cancers (NSCLC) and colorectal carcinomas with wild-type TP53 [49].TMEM16A/ANO1, as a contributor to tumor progression, could inhibit apoptosis via down-regulation of Bim expression [50]. These reports show that Bim may play a critical role in anti-tumor treatment, and may be a new target for clinical cancer therapy strategies.

## Conclusion

Taken together, this is the first study to show that DMAMCL suppressed pediatric cancer in preclinical model. Our results showed that the effect of DMAMCL was partially mediated by Bim induction, and NF-κB pathway and ROS genenration may also contribute to the effect of DMAMCL in RMS cells. Importantly, DMAMCL when combined with chemotherapeutic drugs completely repressed tumor growth in vivo. Given that DMAMCL is already in phase I clinical trials for adult glioma cancer, our preclinical study provides a rational for clinical trial of DMAMCL in RMS pediatric patients.

## Additional files

Additional file 1: **Figure S1.** The effect of VCR or Epirubicin on RMS cells in vitro. (A) RMS cell lines were treated with VCR for 72 h. Cell confluency(%) was calculated using Incucyte Zoom software by phase-contrast images. Each data point represents the mean, SD of triplicate wells. (B) RMS cell lines were treated with Epirubicin for 72 h. Cell confluency(%) was calculated using Incucyte Zoom software by phase-contrast images. Each data point represents the mean, SD of triplicate wells. (TIF 858 kb)

Additional file 2: **Figure S2.** The combination effect of DMAMCL with VCR or Epirubicin on RMS cells in vitro. (A) RD and RH28 cells were treated with DMAMCL and VCR at different concentration in combination for 72 h. Cell survival was evaluated by MTS. Each data point represents the mean, SD of triplicate wells. The combination study was value by CI. CI < 1 indicates synergism, CI = 1 reflects an additive effect, and CI > 1 indicates drug antagonism. (B) RD and RH28 cells were treated with DMAMCL and VCR at different concentration in combination from 0 h to 72 h. Cell confluency(%) was calculated using Incucyte Zoom software by phase-contrast images. Each data point represents triplicate wells. (C) The pictures of RD and RH28 cells were treated with DMAMCL and VCR either alone or in combination for 72 h. (D) RD and RH28 cells were treated with DMAMCL and Epirubicin at different concentration in combination for 72 h. Cell survival was evaluated by MTS. Each data point represents the mean, SD of triplicate wells. The combination study was value by CI. (E) RD and RH28 cells were treated with DMAMCL and Epirubicin at different concentration in combination from 0 h to 72 h. Cell confluency(%) was calculated using Incucyte Zoom software by phase-contrast images. Each data point represents triplicate wells. (F) The pictures of RD and RH28 cells were treated with DMAMCL and Epirubicin either alone or in combination for 72 h. (TIF 3038 kb)

Additional file 3: **FigureS3.** The weight of RMS tumor bearing mice was no change during DMAMCL treatment. RD (*n* = 9), RH18 (*n* = 8), RH30 (*n* = 11), and RH41 (*n* = 9). (TIF 289 kb)

## Abbreviations

ARMS: Alveolar Rhabdomyosarcoma
DMAMCL: Dimethylaminomicheliolide
ERMS: Embryonal Rhabdomyosarcoma
GSL: Guaianolide sesquiterpene lactone
MCL: Micheliolide
PTL: Parthenolide
RMS: Rhabdomyosarcoma
SL: Sesquiterpene lactone
VCR: Vincristine
