# Supplementary figures and images for: The anti-tumor growth effect of a novel agent DMAMCL in rhabdomyosarcoma in vitro and in vivo
Source: J Exp Clin Cancer Res. 2019 Mar 8;38:118. doi: 10.1186/s13046-019-1107-1 (PMC6408795; doi:10.1186/s13046-019-1107-1)

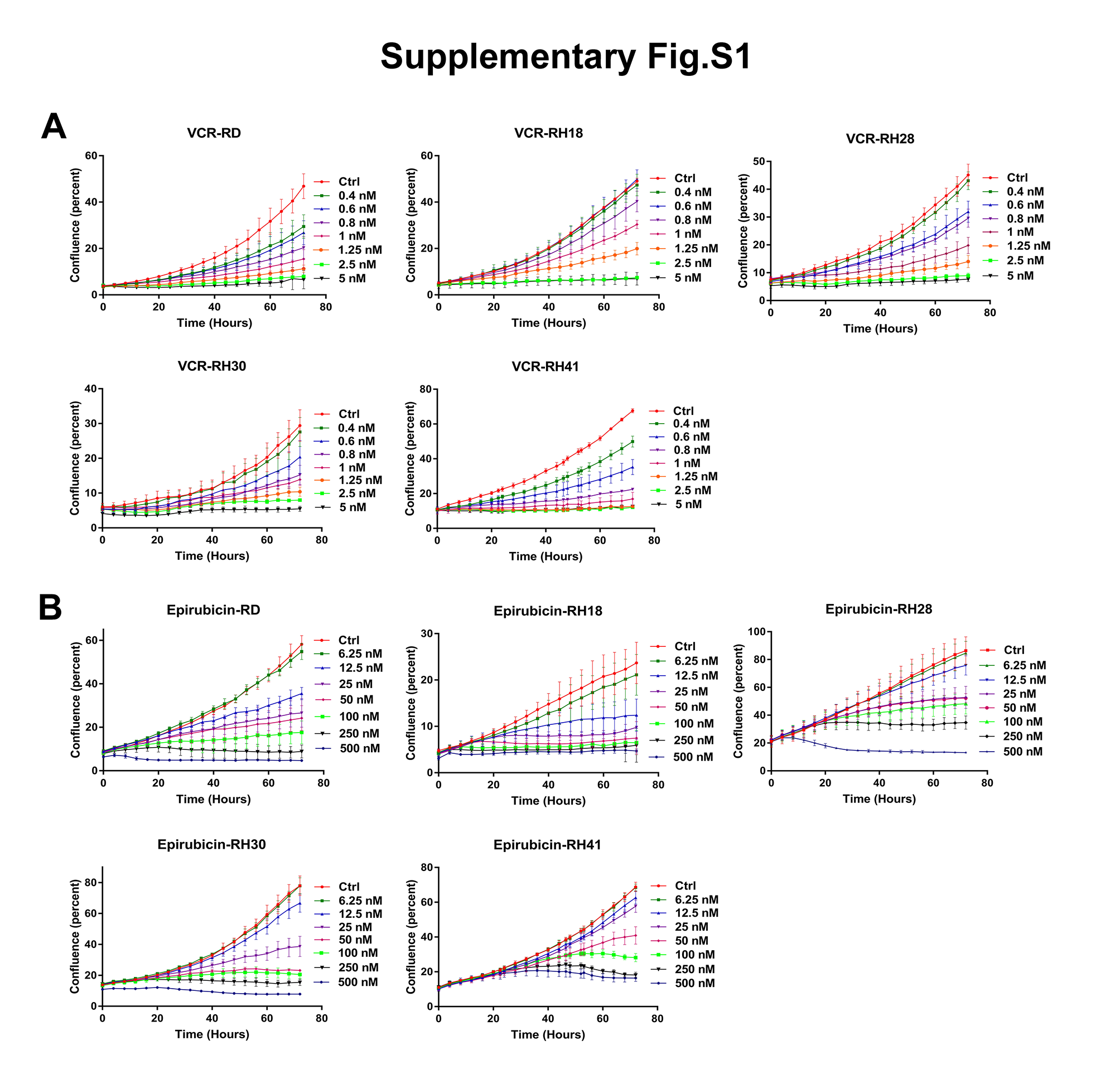

Supplement: Supplementary file 1 — Figure S1. The effect of VCR or Epirubicin on RMS cells in vitro. (A) RMS cell lines were treated with VCR for 72 h. Cell confluency(%) was calculated using Incucyte Zoom software by phase-contrast images. Each data point represents the mean, SD of triplicate wells. (B) RMS cell lines were treated with Epirubicin for 72 h. Cell confluency(%) was calculated using Incucyte Zoom software by phase-contrast images. Each data point represents the mean, SD of triplicate wells. (TIF 858 kb) [file 13046_2019_1107_MOESM1_ESM.tif]

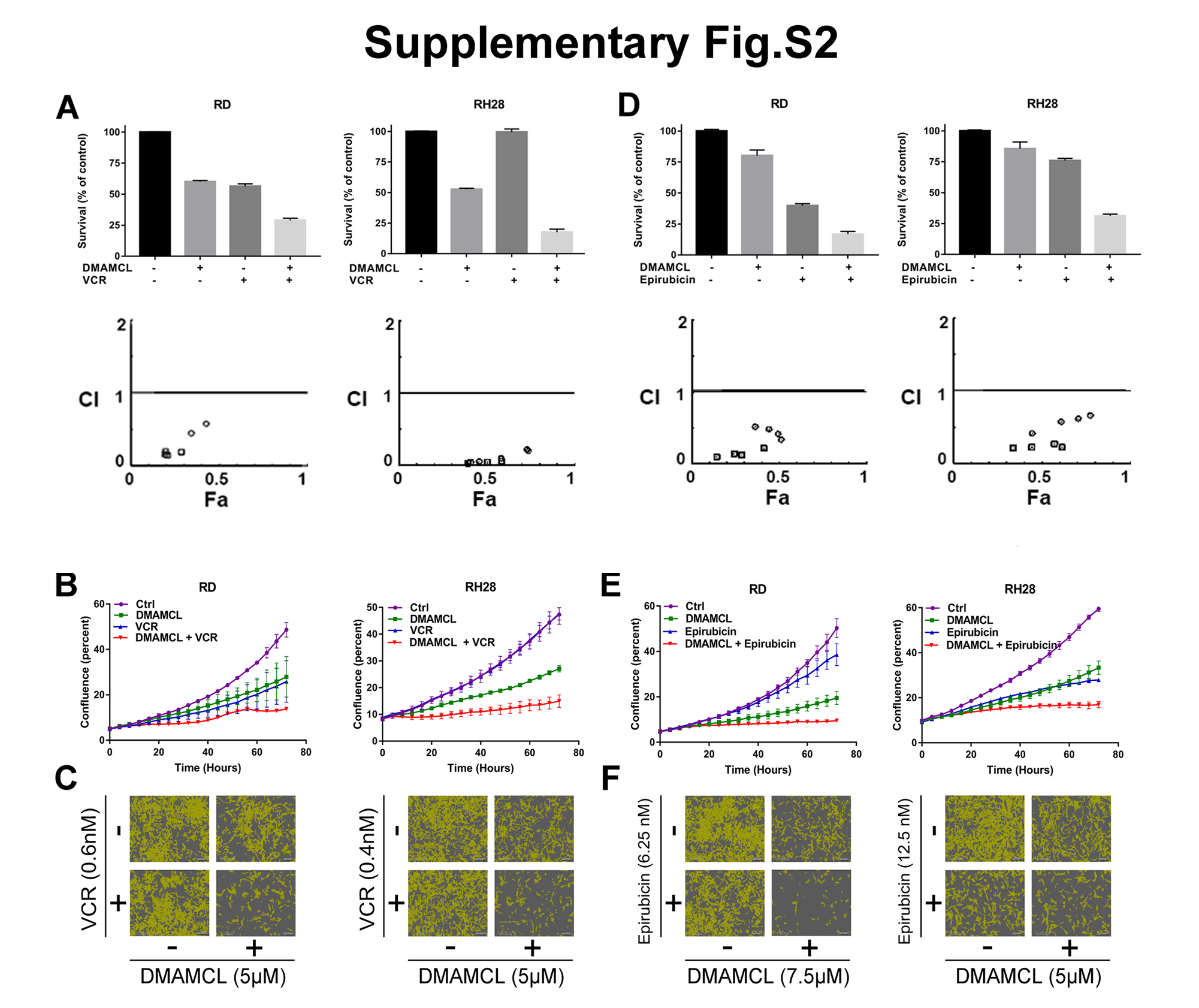

Supplement: Supplementary file 2 — Figure S2. The combination effect of DMAMCL with VCR or Epirubicin on RMS cells in vitro. (A) RD and RH28 cells were treated with DMAMCL and VCR at different concentration in combination for 72 h. Cell survival was evaluated by MTS. Each data point represents the mean, SD of triplicate wells. The combination study was value by CI. CI < 1 indicates synergism, CI = 1 reflects an additive effect, and CI > 1 indicates drug antagonism. (B) RD and RH28 cells were treated with DMAMCL and VCR at different concentration in combination from 0 h to 72 h. Cell confluency(%) was calculated using Incucyte Zoom software by phase-contrast images. Each data point represents triplicate wells. (C) The pictures of RD and RH28 cells were treated with DMAMCL and VCR either alone or in combination for 72 h. (D) RD and RH28 cells were treated with DMAMCL and Epirubicin at different concentration in combination for 72 h. Cell survival was evaluated by MTS. Each data point represents the mean, SD of triplicate wells. The combination study was value by CI. (E) RD and RH28 cells were treated with DMAMCL and Epirubicin at different concentration in combination from 0 h to 72 h. Cell confluency(%) was calculated using Incucyte Zoom software by phase-contrast images. Each data point represents triplicate wells. (F) The pictures of RD and RH28 cells were treated with DMAMCL and Epirubicin either alone or in combination for 72 h. (TIF 3038 kb) [file 13046_2019_1107_MOESM2_ESM.tif]

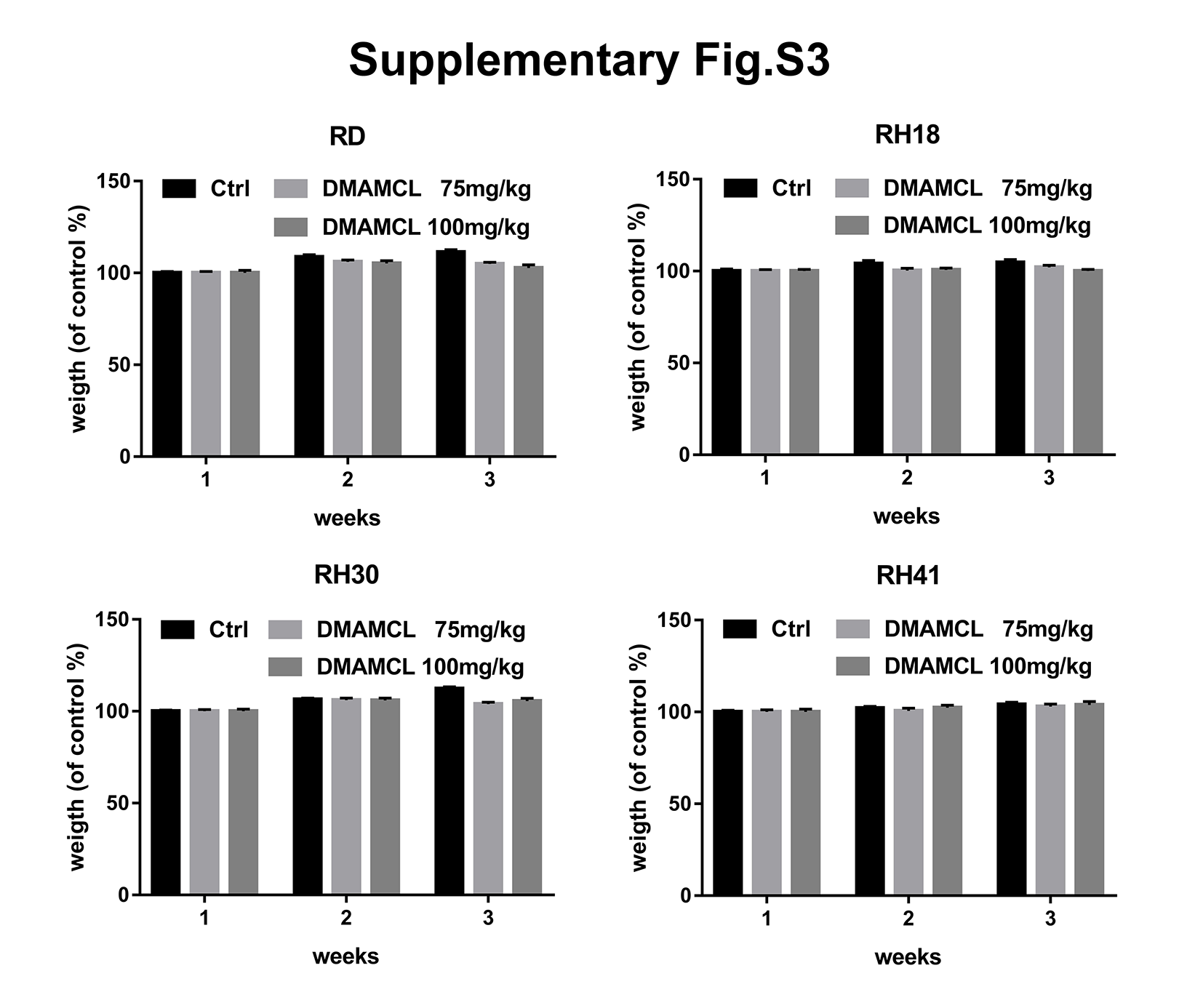

Supplement: Supplementary file 3 — FigureS3. The weight of RMS tumor bearing mice was no change during DMAMCL treatment. RD (n = 9), RH18 (n = 8), RH30 (n = 11), and RH41 (n = 9). (TIF 289 kb) [file 13046_2019_1107_MOESM3_ESM.tif]
